# COVID-19: Growing Health Disparity Gaps and an Opportunity for Health Behavior Discovery?

**DOI:** 10.1089/heq.2020.0026

**Published:** 2020-07-10

**Authors:** Nicole Farmer, Gwenyth R. Wallen, Yvonne Baumer, Tiffany M. Powell-Wiley

**Affiliations:** ^1^ National Institutes of Health, Clinical Center, Bethesda, Maryland, USA.; ^2^ Social Determinants of Obesity and Cardiovascular Risk Laboratory, Cardiovascular Branch, Division of Intramural Research, National Heart, Lung, and Blood Institute, National Institutes of Health, Bethesda, Maryland, USA.; ^3^ Intramural Research Program, National Institute on Minority Health and Health Disparities, Bethesda, Maryland, USA.

**Keywords:** health behavior, psychosocial health, COVID-19, health disparities

## Abstract

Recently, racial and ethnic disparities within the current coronavirus disease-2019 (COVID-19) pandemic at the state level have received attention and notably highlight the ongoing issues surrounding health disparities within the United States. Among the discussions around health disparities lies a discussion on the role of psychosocial stress during this pandemic, especially with broadly applied social distancing and isolation recommendations. In nonpandemic times, psychosocial stressors have a significant association with physiological responses and behavioral responses. Within the current pandemic, increased attention on health-promoting behaviors, such as cooking and physical activity, has occurred. However, based on disparities from structural racism and socioeconomic effects on neighborhood environments, we may see a limiting value to the possible mitigating role of health behaviors within some disparate communities. We present in this perspective that there may be a role for behavioral interventions to mitigate psychosocial stressors and promote health behaviors. It may also be important to consider the use of multilevel behavioral interventions designed in the context of environmental and perceptual barriers during the COIVD-19 pandemic.

Early data at the state level show that disparities within the case and mortality rate from coronavirus disease-2019 (COVID-19) likely exist and disproportionately affect African Americans.^1^ Although not an infection with a predilection for one racial/ethnic group more than another, disparities in cases of COVID-19 may reflect disparities within the health care and economic systems that can lead to inequity of the effects of COVID-19. Reasons for the disparity in COVID-19 affecting African Americans are likely multifactorial, including structural inequities and social injustice, misinformation about infection risk, limited testing availability and health care access, and disproportionate prevalence of preexisting conditions.

The downstream effects of the disparity, however, may be long lasting within the African American community beyond when the pandemic subsides and may mirror health consequences of known social determinants of health. African Americans are not the only racial/ethnic minority community in the United States that may become disproportionately affected by COVID-19.^2^ However, examining the expected disparity among African Americans may shed light on the role of health and socioeconomic inequities as well as identify areas for mitigating the devastating effects of COVID-19 on communities of color. It is important to note that calls for attention to health inequities affecting COVID-19 cases are not limited to the United States as similar concerns have been expressed within China.^3^ In addition, racial/ethnic minorities being disproportionately affected by pandemics are not unique to COVID-19, as during the 2009 H1N1 pandemic, hospitalization rates among minority groups were consistently disproportionate.^4^

As seen in other pandemics within the past century, the current pandemic COVID-19 can create great psychosocial difficulties for affected individuals. Research from prior pandemics has found a wide range of psychosocial impacts, including feelings of helplessness and stigmatization, with negative emotions compounded with the closures of schools and businesses.^5^ Psychosocial studies from the prior severe acute respiratory syndrome (SARS) outbreak between 2002 and 2004 in China showed disparities in psychosocial effects of that pandemic based on socioeconomic status.^5,6^ A unique factor that may lead to psychosocial disturbance from this pandemic versus others, such as Ebola, is that for COVID-19, it is difficult and challenging to detect and distinguish the infection from benign conditions. This may place additional psychosocial stress on individuals because social isolating recommendations such as distancing or self-quarantining may be more broadly applied.

Most pandemics in the past century have received limited study by behavioral scientists.^7,8^ For those studies that have occurred, attention has rightfully focused on the effect of psychological stressors on preventive or infection-related behaviors. In addition, less attention has been given to the role of health-promoting behaviors during pandemics.

In the United States, the biased social construct of race^9^ has led to a historical legacy of African Americans being the recipients of prejudicial, discriminatory, and racially based exclusionary practices. The ultimate culmination of these practices has led to many African Americans having life experiences rooted in racial injustice at individual, structural, and institutional levels. The consequences of these racial injustice experiences may impact and constrain health. Experiences with receiving care that is driven by implicit racial biases, explicit use of race in medical decision making, or perceptions of differential health care services have led to significant barriers surrounding trust when it comes to interactions in health care settings.^10–12^ Also, repeated exposure to microaggressions, discrimination, and racist practices, especially those of social exclusion, can stimulate physiological stress responses that, when occurring daily, or when occurring in an unpredictable pattern, can be akin to chronic stressors.

In addition, discriminatory and exclusionary practices have led to significant racial differences and inequities. Predominately, African American communities have fewer economic opportunities, including less home ownership and a disproportionate poverty concentration.^13^ Consequences of these neighborhood environments can directly impact physical and mental health: less access to foods identified to prevent chronic diseases,^14^ increased exposure to violence,^15,16^ and less access to green space,^17^ a known mitigating factor of psychosocial stress. As a result, chronic physiological, psychosocial, and environmental stressors may overburden African Americans and, in an inequitable manner, influence poorer health outcomes.

Furthermore, immune responses to adverse psychosocial and environmental conditions may contribute to an increased risk of COVID-19 morbidity and mortality among African Americans.^18,19^ Specifically in relation to COVID-19, interleukin-6 (IL-6), an inflammatory cytokine associated with chronic stress and with demonstrated racial/ethnic differences, appears to be a key mechanism involved in COVID-19 disease severity. In particular, an association between the sympathetic nervous system hormone adrenaline and IL-6 is found in conditions with increased COVID-19 mortality and underlying chronic diseases disproportionately found among African Americans, including cardiovascular disease,^20^ diabetes,^21^ and hypertension.^22^ Novel or unique stressors, as may be experienced during a pandemic, can lead to exaggerated sympathetic responses.^23^ Therefore, there may be a key reason to examine a role for psychosocial factors related to African Americans during this pandemic.

As already stated, a key consideration within the pandemic is the role of health behaviors and how they impact psychosocial health. Health-promoting behaviors, those behaviors actively done by an individual for maintenance or improvement of one's health or to maintain a healthy lifestyle, typically include physical activity, sleep, and dietary behaviors. In nonpandemic situations, psychosocial factors are noted to have a significant association with health-promoting behaviors through physiological (neuroendocrine, cardiovascular, inflammatory, and immune effects) and behavioral responses.^24–26^ Hence these behaviors may help to restore some biological and psychological mechanisms negatively affected by psychosocial stress. But engagement in these behaviors may be encouraged or hindered by psychosocial stressors during a pandemic. In fact, a recent web-based survey involving the general population in China found a significant association with anxiety around COVID-19 and sleep disturbances.^27^ Since the start of the COVID-19 pandemic, two health-promoting behaviors have received specific attention: cooking and physical activity. Although there is emerging interest on the role of cooking in psychosocial health,^28^ in particular, physical activity may lead to positive effects on lowering IL-6 levels^29^ and on immunity and inflammation even in non-COVID-19 viral respiratory infections.^30^ Examination of reports on these and other COVID-19–related health behaviors may provide areas of investigation that are key to understanding the impact of this pandemic on health-promoting behaviors. For example, are socioeconomic factors, time availability, or psychosocial factors driving or limiting participation in healthy behaviors during the COVID-19 outbreak? What is the significance of having a history of health-promoting behavior before the COVID-19 pandemic? Do motivation and self-efficacy play a role in engagement in the healthy behavior during prolonged social distancing? And are interpersonal psychosocial changes occurring as a result of either starting or increasing the behavior? Most importantly, once the COVID-19 social restrictions are lifted, will this health-promoting behavior be maintained or cease, and what are the significant social determinants of such actions?

Identifying the role of COVID-19 on health behaviors could be especially important for certain populations that are affected by health disparities, such as African Americans. As COVID-19–related social distancing and quarantining conditions may influence involvement in health behaviors, there may be a limit to engaging in these behaviors among these communities due to known social determinants of health, such as type of employment, food access, and perceived or objective neighborhood environment conditions. Recognizing the connection of psychosocial experiences to broader social and environmental conditions, in 2005, the WHO Commission on Social Determinants of Health recognized psychosocial factors as social determinants to health outcomes.^31^ These factors included social support, social exclusion, and psychological stress. This connection provides a context for understanding the importance of targeting different areas that influence psychosocial health and related health behaviors. We suggest work is urgently needed to not only elucidate mechanisms by which these psychosocial factors may be influenced by other social determinants such as an individual's physical environment, but also to develop interventions that might effectively reduce psychosocial stressors during the COVID-19 pandemic for the most vulnerable populations. For example, mindfulness-based stress-reduction interventions are shown to decrease blood pressure readings among low-income African American adults.^32^ And intervention studies shown to increase self-compassion^33^ and increase social cohesion^34^ may lead to decreased IL-6 levels. Notably, social cohesion and activities that promote such cohesion, including engaging in church services, are recognized psychosocial coping factors among African Americans,^35,36^ even within the context of psychosocially stressful events.^37^ Thus, these intervention approaches may allow for the leveraging of existing coping skills and resilience factors in African American communities. The use of sociocultural resilience skills may lead to compounded benefits through reinforcing a positive collective identity. The resultant social capital from this may help to propagate further the use of these skills among individuals.

Multilevel interventions based on socioecological models including levels for institutional/policy, neighborhood, interpersonal relationships, and individual factors may be especially important to consider.^38^ These interventions are uniquely effective at addressing minority health and disparities by providing interventions based on the interconnected levels involved in creating disparities. Examples of how multilevel interventions may be useful during this pandemic to address psychosocial health and health-promoting behaviors may include health communication and messaging interventions (institutional and societal levels); promotion and identification of locations within an area to engage in social distancing physical activities (neighborhood levels); family-based interventions around instrumental or emotional support (interpersonal level); and psychological interventions on perception and self-awareness of behaviors (individual level). Disciplines involved in these interventions may include health behavior research, positive psychology, and social science. Lastly leveraging technology, especially accessibility of smartphone-based platforms, may help to advance the use of multilevel interventions during the current pandemic.

In conclusion, to strategically and effectively address health disparity outcomes during the COVID-19 pandemic will require local, state, and federal public health resources with focused health behavior and public health research to address issues related to specific risks among communities of color, specifically African Americans. We suggest that deliberate and timely attention to psychosocial health among African Americans during the pandemic may also offer opportunities to mitigate expected disproportionate effects of COVID-19, may help to highlight the role of certain environmental factors on psychosocial health, and may additionally introduce novel health-promoting strategies to individuals, families, and their communities.

## Abbreviations Used

COVID-19: coronavirus disease-2019
IL-6: interleukin-6

## References

[1] Prorepublia.com Available at https://www.propublica.org/article/early-data-shows-african-americans-have-contracted-and-died-of-coronavirus-at-an-alarming-rate Accessed 43, 2020

[2] The Atlantic. Available at https://www.theatlantic.com/ideas/archive/2020/04/coronavirus-exposing-our-racial-divides/609526/ Accessed 45, 2020

[3] Wang Z, Tang K Combating COVID-19: health equity matters. Nat Med. 2020;26:458 32284617 10.1038/s41591-020-0823-6

[4] CDC. 2010 Centers for Disease Control and Prevention, “Information on 2009 H1N1 Impact by Race and Ethnicity.” Available at www.cdc.gov/h1n1flu/race_ethnicity_qa.htm, last updated February 24, 2010 2:00 PM ET. Accessed 47, 2020

[5] Wang C, Ran R, Wan X, et al. Immediate Psychological responses and associated factors during the initial stage of the 2019 Coronavirus disease epidemic among the general population in China. Int J Environ Res Public Health. 2020;17:1729 10.3390/ijerph17051729 PMC7084952 32155789

[6] Morganstein J, Fullerton C, Ursano R, et al. Pandemics: health care emergencies. In: Textbook of Disaster Psychiatry. Edited by Ursano R, Fullerton C, Weisaeth L, Raphel B. Cambridge: Cambridge University Press, 2017, pp. 270–284

[7] Rubin GJ, Wessely S The psychological effects of quarantining a city. BMJ. 2020;368:m313 31992552 10.1136/bmj.m313

[8] Brooks SK, Webster RK, Smith LE, et al. Psychological impact of quarantine and how to reduce it: a rapid review of the evidence. Lancet. 2020;395:P912–P992 10.1016/S0140-6736(20)30460-8 PMC7158942 32112714

[9] Smedley A, Smedley BD Race as biology is fiction, racism as a social problem is real: anthropological and historical perspectives on the social construction of race. Am Psychol. 2005;60:16–26 15641918 10.1037/0003-066X.60.1.16

[10] Burgess DJ Are providers more likely to contribute to healthcare disparities under high levels of cognitive load? How features of the healthcare setting may lead to biases in medical decision making. Med Decis Making. 2010;30:246–257 19726783 10.1177/0272989X09341751 PMC3988900

[11] Armstrong K, Ravenell KL, McMurphy S, et al. Racial/ethnic differences in physician distrust in the United States. Am J Public Health. 2007;97:1283–1289 17538069 10.2105/AJPH.2005.080762 PMC1913079

[12] Snipes SA, Sellers SL, Tafawa AO, et al. Is race medically relevant? A qualitative study of physicians’ attitudes about the role of race in treatment decision-making. BMC Health Serv Res. 2011;11:183 21819597 10.1186/1472-6963-11-183 PMC3167748

[13] Firebaugh G, Acciai F For blacks in America, the gap in neighborhood poverty has declined faster than segregation. Proc Natl Acad Sci U S A. 2016;113:13372–13377 27821759 10.1073/pnas.1607220113 PMC5127296

[14] Morland K, Wing S, Diez Roux A, et al. Neighborhood characteristics associated with the location of food stores and food service places. Am J Prev Med. 2002;22:23–29 11777675 10.1016/s0749-3797(01)00403-2

[15] Browning CR, Calder CA, Ford JL, et al. Understanding racial differences in exposure to violent areas: integrating survey, smartphone, and administrative data resources. Ann Am Acad Pol Soc Sci. 2017;669:41–62 28845047 10.1177/0002716216678167 PMC5567748

[16] Goldmann E, Aiello A, Uddin M, et al. Pervasive exposure to violence and posttraumatic stress disorder in a predominantly African American Urban Community: The Detroit Neighborhood Health Study. J Trauma Stress. 2011;24:747–751 22144187 10.1002/jts.20705 PMC4433006

[17] Wen M, Zhang X, Harris CD, et al. Spatial disparities in the distribution of parks and green spaces in the USA. Ann Behav Med. 2013;45 (Suppl 1):S18–S27 23334758 10.1007/s12160-012-9426-x PMC3590901

[18] Bagby SP, Martin D, Chung ST, et al. From the outside in: biological mechanisms linking social and environmental exposures to chronic disease and to health disparities. Am J Public Health. 2019;109(S1):S56–S63 30699032 10.2105/AJPH.2018.304864 PMC6356139

[19] Baumer Y, Gutierrez-Huerta CA, Saxena A, et al. Immune cell phenotyping in low blood volumes for assessment of cardiovascular disease risk, development, and progression: a pilot study. J Transl Med. 2020;18:29 31952533 10.1186/s12967-020-02207-0 PMC6966880

[20] Piira OP, Miettinen JA, Hautala AJ, et al. Physiological responses to emotional excitement in healthy subjects and patients with coronary artery disease. Auton Neurosci. 2013;177:280–285 23916871 10.1016/j.autneu.2013.06.001

[21] Asadikaram G, Ram M, Izadi A, et al. The study of the serum level of IL-4, TGF-beta, IFN-gamma, and IL-6 in overweight patients with and without diabetes mellitus and hypertension. J Cell Biochem. 2019;120:4147–4157 30260038 10.1002/jcb.27700

[22] Carnagarin R, Matthews V, Zaldivia MTK, et al. The bidirectional interaction between the sympathetic nervous system and immune mechanisms in the pathogenesis of hypertension. Br J Pharmacol. 2019;176:1839–1852 30129037 10.1111/bph.14481 PMC6534787

[23] Uschold-Schmidt N, Nyuyki KD, Fuchsl AM, et al. Chronic psychosocial stress results in sensitization of the HPA axis to acute heterotypic stressors despite a reduction of adrenal in vitro ACTH responsiveness. Psychoneuroendocrinology. 2012;37:1676–1687 22444976 10.1016/j.psyneuen.2012.02.015

[24] Segerstrom SC, Miller GE Psychological stress and the human immune system: a meta-analytic study of 30 years of inquiry. Psychol Bull. 2004;130:601–630 15250815 10.1037/0033-2909.130.4.601 PMC1361287

[25] Uchino BN Social support and health: a review of physiological processes potentially underlying links to disease outcomes. J Behav Med. 2006;29:377–387 16758315 10.1007/s10865-006-9056-5

[26] Goldstein DS, McEwen B Allostasis, homeostats, and the nature of stress. Stress. 2002;5:55–58 12171767 10.1080/102538902900012345

[27] Haung Y, Zhao N Generalized anxiety disorder, depressive symptoms and sleep quality during COVID-19 epidemic in China: a web-based cross-sectional survey. medRxiv. 2020;DOI: 10.1101/2020.02.19.20025395. PMC7152913 32325383

[28] Farmer N, Touchton-Leonard K, Ross A Psychosocial benefits of cooking interventions: a systematic review. Health Educ Behav. 2018;45:167–180 29121776 10.1177/1090198117736352 PMC5862744

[29] Starkweather AR The effects of exercise on perceived stress and IL-6 levels among older adults. Biol Res Nurs. 2007;8:186–194 17172317 10.1177/1099800406295990

[30] Nieman DC, Wentz LM The compelling link between physical activity and the body's defense system. J Sport Health Sci. 2019;8:201–217 31193280 10.1016/j.jshs.2018.09.009 PMC6523821

[31] CSDH. Closing the gap in a generation: health equity through action on the social determinants of health. Final Report of the Commission on Social Determinants of Health. Geneva: World Health Organization, 2008 10.1016/S0140-6736(08)61690-6 18994664

[32] Palta P, Page G, Piferi RL, et al. Evaluation of a mindfulness-based intervention program to decrease blood pressure in low-income African-American older adults. J Urban Health. 2012;89:308–316 22302233 10.1007/s11524-011-9654-6 PMC3324609

[33] Breines JG, Thoma MV, Gianferante D, et al. Self-compassion as a predictor of interleukin-6 response to acute psychosocial stress. Brain Behav Immun. 2014;37:109–114 24239953 10.1016/j.bbi.2013.11.006 PMC4311753

[34] Neergheen VL, Topel M, Van Dyke ME, et al. Neighborhood social cohesion is associated with lower levels of interleukin-6 in African American women. Brain Behav Immun. 2019;76:28–36 30686334 10.1016/j.bbi.2018.10.008 PMC6370481

[35] Utsey SO, Giesbrecht N, Hook J, et al. Cultural, sociofamilial, and psychological resources that inhibit psychological distress in African Americans exposed to stressful life events and race-related stress. J Counsel Psychol. 2008;55:49–62

[36] Brenner AB, Diez-Roux AV, Gebreab SY, et al. The epidemiology of coping in African American adults in the Jackson heart study. J Racial Ethn Health Disparities. 2018;5:978–994 29218498 10.1007/s40615-017-0445-y PMC6060024

[37] Hackbarth M, Pavkov T, Wetchler J, et al. Natural disasters: an assessment of family resiliency following Hurricane Katrina. J Marital Fam Ther. 2012;38:340–351 22512296 10.1111/j.1752-0606.2011.00227.x

[38] Agurs-Collins T, Persky S, Paskett ED, et al. Designing and assessing multilevel interventions to improve minority health and reduce health disparities. Am J Public Health. 2019;109 (S1):S86–S93 30699029 10.2105/AJPH.2018.304730 PMC6356127

